# Aggregation and interfacial phenomenon of amphiphilic drug under the influence of pharmaceutical excipients (green/biocompatible gemini surfactant)

**DOI:** 10.1371/journal.pone.0211077

**Published:** 2019-02-06

**Authors:** Malik Abdul Rub

**Affiliations:** Chemistry Department, Faculty of Science, King Abdulaziz University, Jeddah, Saudi Arabia; Brandeis University, UNITED STATES

## Abstract

In the current study, we have examined the interaction amongst an antidepressant drug amitriptyline hydrochloride (AMH) and ethane-1, 2-diyl bis(*N*,*N*-dimethyl-*N*-cetylammoniumacetoxy) dichloride (16-E2-16, a green gemini surfactant) through tensiometric and fluorimetric techniques in aqueous/electrolyte/urea solutions. Significant variations are observed in the various evaluated parameters in the present study. Gemini 16-E2-16 has outstanding surface properties along with a much lower *cmc* value, demonstrating very little toxicity as well as considerable antimicrobial activity. The *cmc* values of mixtures decrease through increase in mole fraction (*α*_1_) of 16-E2-16, which specifies the nonideality of the solution mixtures, along with demonstrating the occurrence of mixed micellization too. Negative *β*^Rub^ values signify on the whole attractive force of interaction between constituents of mixed micelles. Owing to the incidence of electrolyte NaCl (50 mmol.kg^–1^), lowering of the micelles’ surface charge happens, resulting in aggregation taking place at lower concentration while the presence of urea (NH_2_CONH_2_) halts micellization taking place, which means the *cmc* value increases in the attendance of urea. The $\Delta G_{m}^{o}$ values for all systems were negative along with the presence of electrolyte/urea. The excess free energy (*G*_ex_) of studied mixed systems was also estimated and found to be negative for all the systems. Using the fluorescence quenching method, the micelle aggregation number (*N*_agg_) was evaluated and it was found that the contribution of gemini surfactant was always more than that of the AMH and their value enhances in the existence of electrolyte while decreasing in the attendance of NH_2_CONH_2_ in the system. In addition, other fluorescence parameters such as micropolarity (*I*_1_/*I*_3_), dielectric constant (*D*_exp_) as well as Stern–Volmer binding constants (*K*_sv_) of mixed systems were evaluated and the results showed the synergistic performance of the AMH + 16-E2-16 mixtures. Along with tensiometric and fluorimetric techniques, FT-IR spectroscopy was also engaged to reveal the interaction among constituents.

## Introduction

Amphiphiles such as surfactants have been receiving consideration due to their extraordinary properties as well as numerous overlay uses as a part of pharmaceutics, drug delivery, emulsification, nanomaterial preparation, vesicle development, oil recuperation and so on [1–5]. Above a particular concentration, amphiphile molecules form micelles in aqueous as well as nonaqueous solution [5–8]. The concentration beyond which the formation of micelles starts is labeled the critical micelle concentration (*cmc*) [5–8]. It is important for most reasonable applications to select a mixture of amphiphiles to achieve the desired characteristics [5]. Mixed amphiphile mixtures are also be helpful for the environment because the amount of amphiphiles discharged and therefore their impact could probably be reduced considerably [5,9]. In the pharmaceutical industry, the absorption of several drugs in human beings is enhanced by way of micelles [10].

The name gemini surfactants (the name gemini was created by Menger [11–13]) was assigned to long hydrophobic amphiphile molecules acquiring, consecutively, an elongated hydrocarbon tail keeping a charged head group, an inflexible spacer, another charged head group, along with one more hydrocarbon chain [11–13]. Both hydrocarbon chains having alike charged groups can be connected directly via a spacer, on the other hand, both alike amphiphiles are bonded halfway. Currently, gemini surfactants, as a result of their exceptional along with fascinating features are receiving considerable significance in scientific culture [13]. As stated above, gemini's are made out of two hydrophobic chains connected on or close to the head portions through a small, elongated, inflexible or stretchy spacer [5]. They have advanced physicochemical performance, for instance brilliant surface-active assets, excellent surface tension decreasing capability, extraordinarily small *cmc*, additional viscoelasticity as well as high solubilization potential as compared with the traditional surfactants keeping a single hydrophobic portion as well as a single head group in a single monomer [14,15]. These interesting characters of geminis together with the growing claim for high activity surfactants formulate them with exceptional significance for pharmaceutical purposes. In spite of the above excellent characteristics, the majority are nonbiodegradable and for this reason cause environmental problems. As a result, in the recent past the cleavable surfactant (traditionally along with gemini) is of great attention. A distinct cleavable amphiphile such as 16-E2-16 (ethane-1, 2-diyl bis(*N*,*N*-dimethyl-*N*-cetylammoniumacetoxy) dichloride) is important due to the attendance of a feeble diester (E2, a hydrophilic moiety) linkage as a spacer; for that reason, formulates them biodegradable, cleavable as well as lower *cmc* (1000-folds below the traditional surfactants). In addition, the presence of a diester in gemini improves their aggregation conduct through hydrogen bonding involvement. As a result, it is an appropriate nominee for examining their interaction by means of additives. Numerous studies on mixed micellization behavior of basic polymethylene spacer kind gemini surfactant through amphiphiles (traditional surfactant, drugs etc.) are available [16–18], but studies on the interaction between cleavable cationic geminis and amphiphiles such as drugs are much less [19]. Therefore, this gemini is a noteworthy unit to be in use for more research. Currently employed gemini surfactants are better substitutes than other classes of gemini surfactants [20,21]. By way of this perspective, numerous surfactants have been synthesized recently having polar bonds and they are exceedingly soluble, simply hydrolyzable, as well as degradable [5,13,14]. The spacer having ester bond makes currently employed gemini additionally cleavable, eco-friendly; in addition, this surfactant has lower marine toxicity than the other surfactants [20,21].

**Fig 1:**
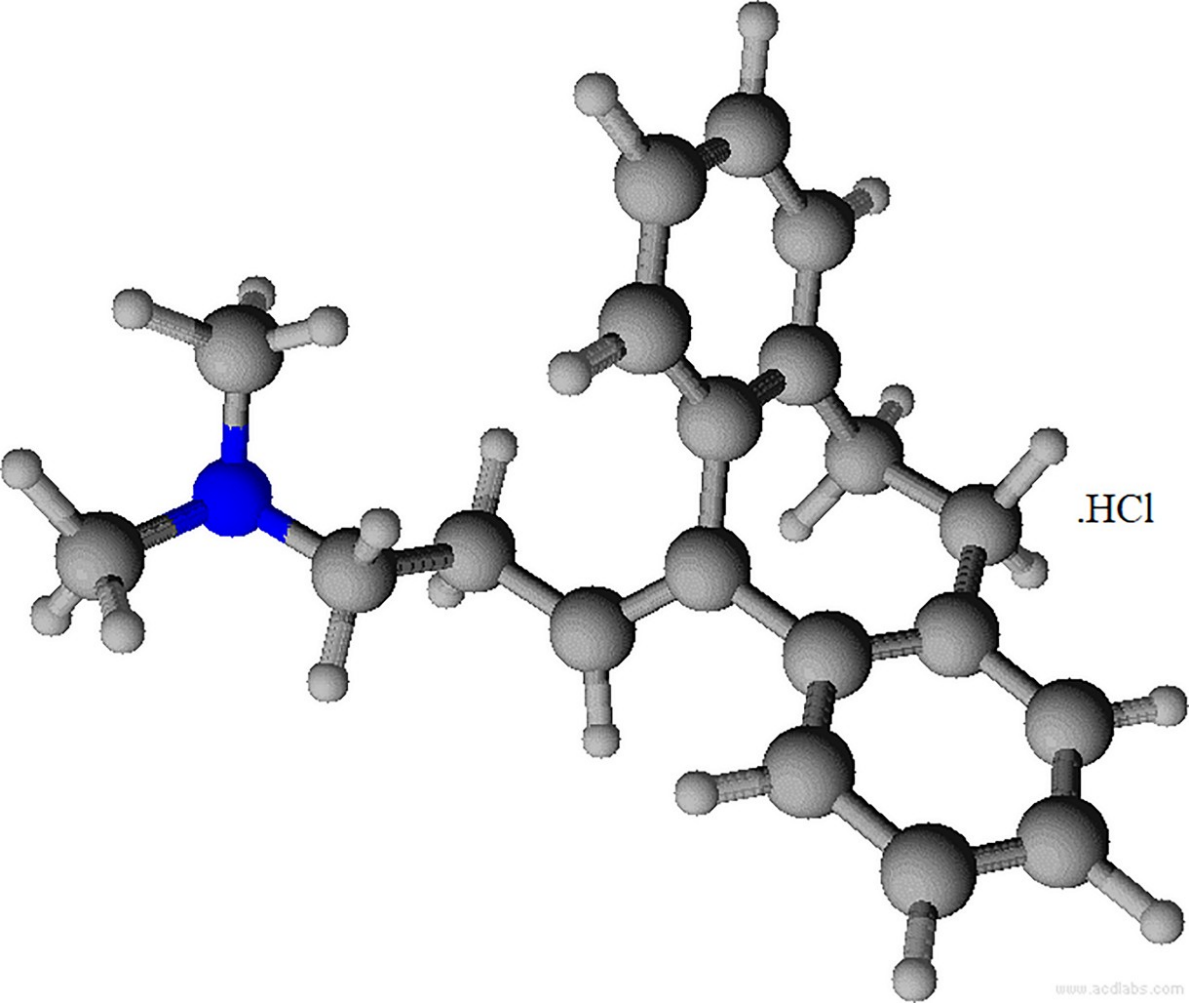
Molecular structure of amitriptyline hydrochloride (AMH).

A pharmacologically active drug amitriptyline hydrochloride (AMH) is employed as an antidepressant and it holds a unbendable tricyclic ring as well as a tiny alkylamine chain having a terminal nitrogen particle, furthermore, by reason of the occurrence of hydrocarbon side chain this class of drug forms micelles independently, akin to usual surfactants (Fig 1) [1,22]. AMH is employed to cure the expression of depression. Recently, the study of the drug-additive interaction has received increased attention because of the extensive application of these systems in the applied field. Maximum drugs facing a lot of unwanted outcomes along with their actual effects. These undesirable effects of drugs are lessened if drugs are employed via an apposite drug shipper, such as green/biocompatible gemini.

**Fig 2:**
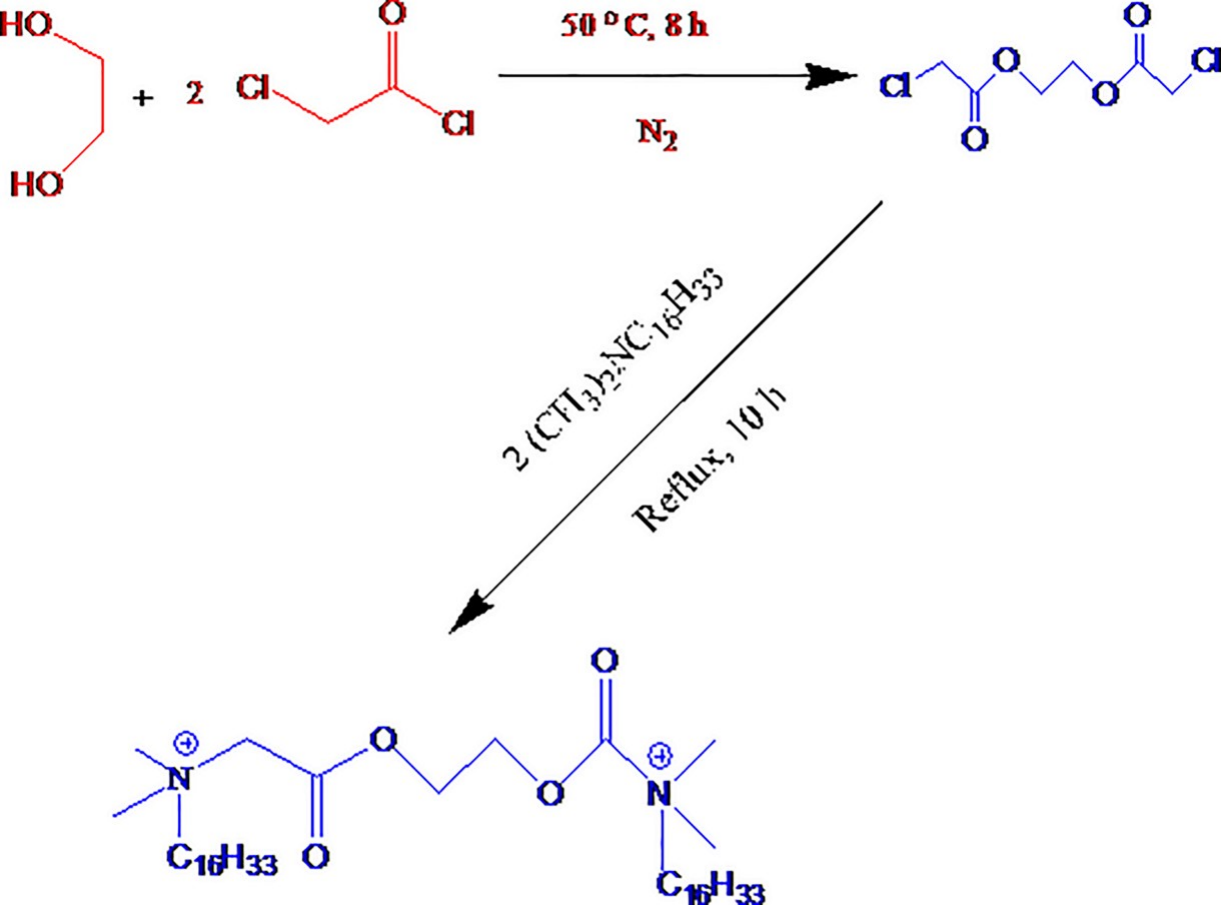
Synthesis route of the biodegradable cationic gemini surfactants (16-E2-16).

To our knowledge, work on physicochemical characterization of conventional surfactant and amphiphilic drug mixtures has been done exhaustively earlier; but, the work concerning aggregation, adsorption along with microstructural phenomena of gemini surfactants and drug mixtures is up to now rare [23,24]. Taking into consideration the above facts along with also our curiosity in recent innovative surfactants, in the current study, we synthesized an ester-functionalized green gemini surfactant, 16-E2-16 (Fig 2) along with examining their interaction through amitriptyline hydrochloride (AMH). 16-E2-16 encloses a feeble diester linkage that makes them additionally noteworthy regarding lower *cmc*. To our information, no description of the interaction of 16-E2-16–AMH mixtures has been given earlier. In the current study, we have discussed the aggregation along with air–water surface possessions in addition additional parameters of mixed systems of both constituents. Tensiometric, fluorimetric and FT-IR techniques were engaged to evaluate the interaction of AMH–16-E2-16 mixed systems in the absence plus occurrence of additives [25–28]. The chief objective of this investigation is to elucidate the microstructure of the gemini surfactant micelles as well as to evaluate the ability of these aggregates to incorporate ionic drug.

## Experimental procedure

### Materials

All preliminary materials engaged in the current study are analytical rating and utilized as achieved. Table 1 provides details about the chemicals used including the CAS numbers, sources, and mass fraction purity. Deionized double distilled water (DDW) with conductivity amongst (1 to 6) x 10^−6^ S cm^–1^ was utilized throughout the study for the preparation of the reserve solution of amitriptyline hydrochloride (AMH) along with 16-E2-16 both in the absence as well as existence of NaCl/urea.

**Table 1:** The origin and purity of the molecules utilized in the current work.

Chemical name	Source	CAS number	Purification methods	Mass fraction purity	Analytic methods
Amitriptyline hydrochloride (AHC)	Sigma (USA)	549-18-8	Vacuum drying	≥ 0.98	TLC^a^
16-E2-16	Synthesized in lab	-	Recrystallization in ethyl acetate-ethanol and vacuum drying	0.99	NA
NaCl	BDH (England)	7647-14-5	Vacuum drying	0.98	NA
Urea	Sigma (Germany)	57-13-6	Vacuum drying	0.98	HPLC^b^
Pyrene (PR)	Sigma (USA)	129-00-0	Vacuum drying	0.99	NA
Cetylpyridinium chloride monohydrate (CC)^c^	Merck (Germany)	6004-24-6	Vacuum drying	-	NA

^a^TLC, thin layer chromatography

^b^HPLC, high performance liquid chromatography (provided by supplier).

^c^Anhydrous salt attained after drying the cetylpyridinium chloride hydrate revealed in the table. The H_2_O content in the hydrated salt evaluated by means of Karl-Fisher analysis was obtained to be below 100 ppm.

#### Synthesis of green gemini surfactant

The currently employed biodegradable green gemini surfactant, 16-E2-16, is cationic in nature and was synthesized in our laboratory by means of a known method and the aspects are accounted in the literature (Fig 2) [29]. The synthesized compound was recrystallized through suitable solvent mixed systems of ethyl acetate and ethanol. ^1^H NMR as well as FT-IR were employed for characterization of the final compound and the obtained data were achieved to be in fine conformity through the earlier accounted values [29,30]. Moreover the purity of prepared 16-E2-16 gemini surfactant was validated as a result of the no minimum in surface tension (*γ*) against log concentration of amphiphile plot [5].

### Method

#### Surface tension determinations

**Fig 3:**
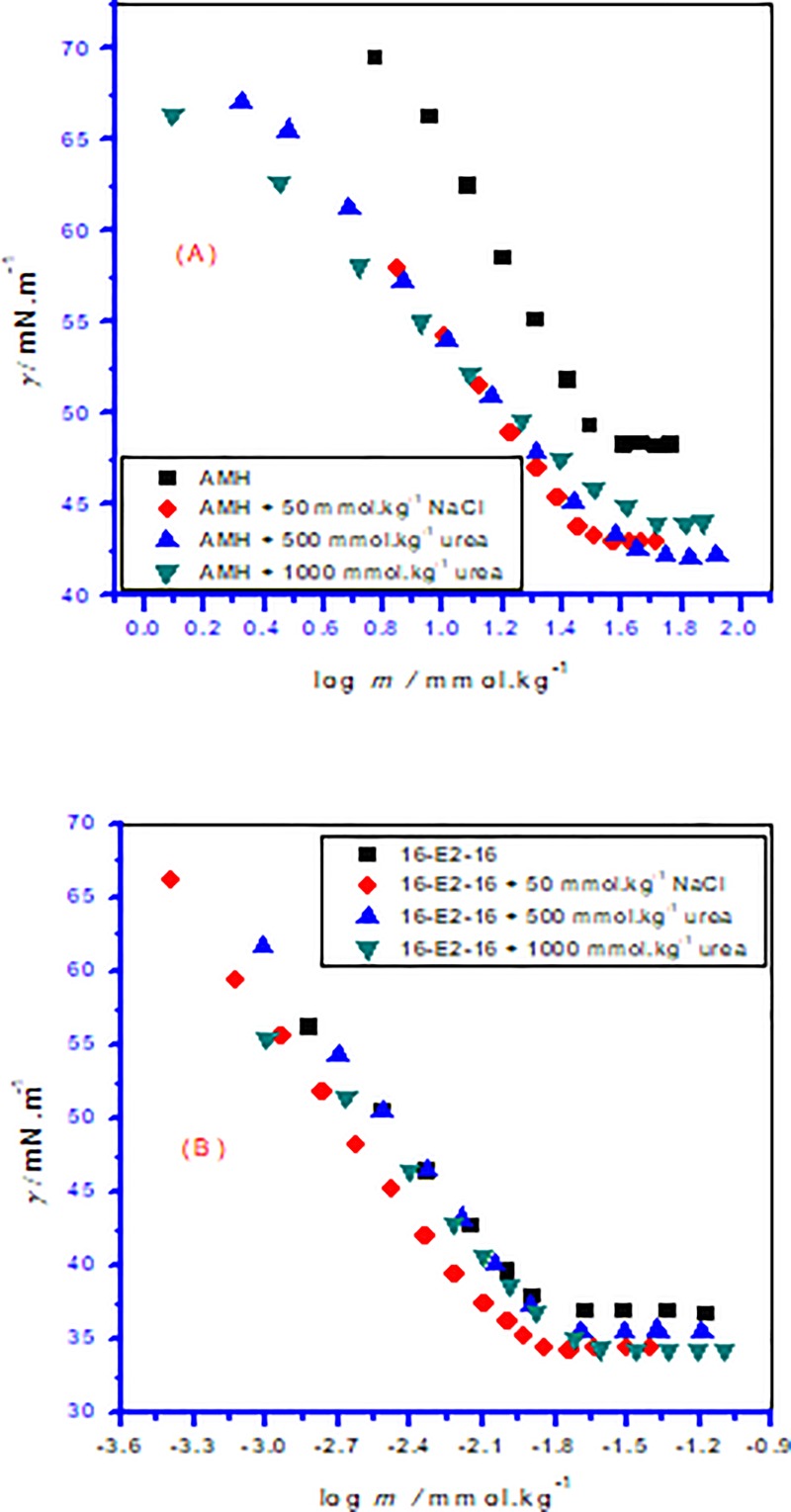
Surface tension (*γ*) versus concentration (*m*) isotherms for pure amphiphiles ((A) AMT and (B) 16-E2-16) in different media at 298.15 K.

**Fig 4:**
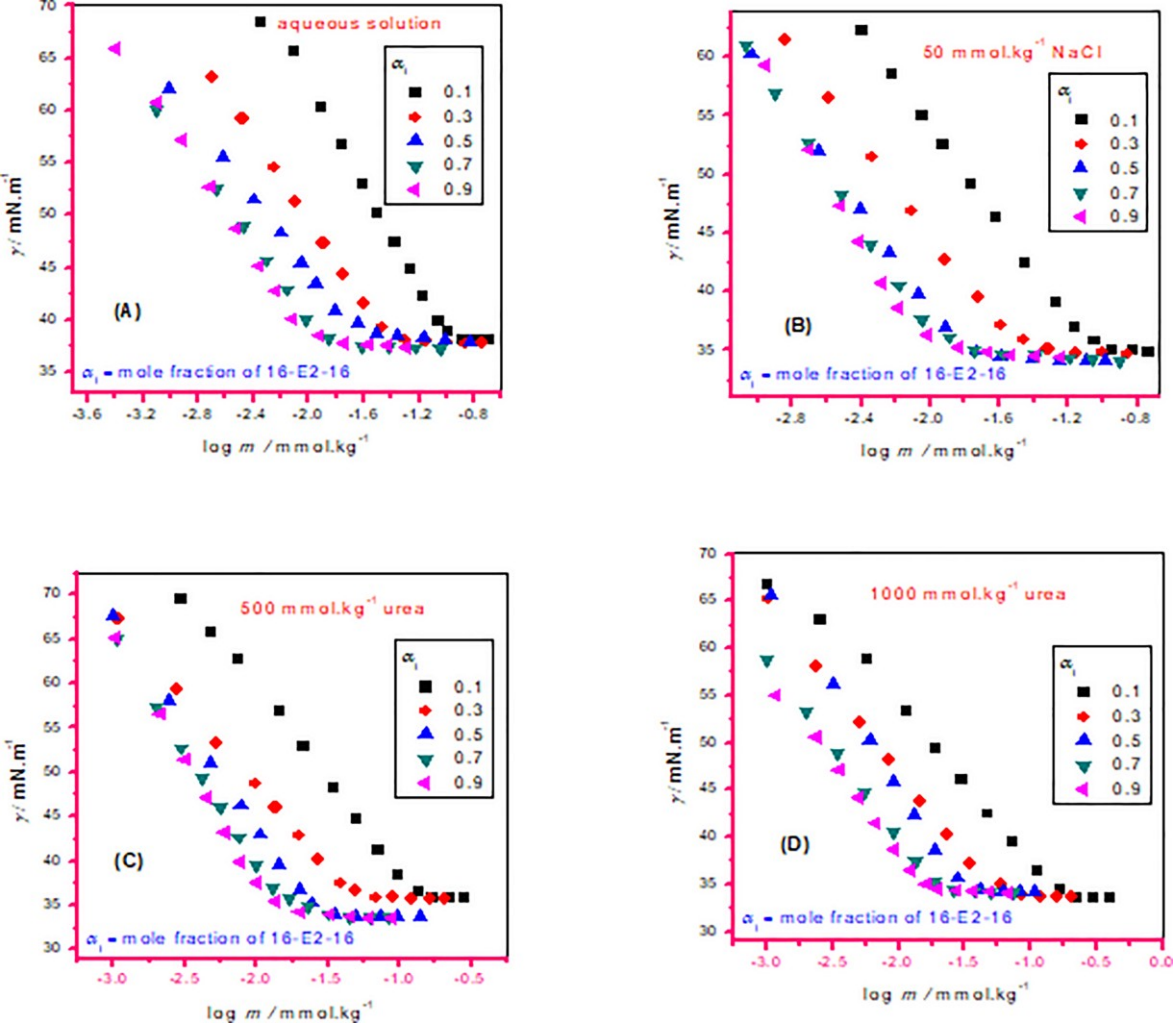
Surface tension (*γ*) with concentration (*m*) isotherms for AMH-16-E2-16 mixture in different ratio (different mole fraction of 16-E2-16 (*α*_1_)): (A) aqueous solution, (B) 50 mmol∙kg^-1^ NaCl, (C) 500 mmol∙kg^-1^ NH_2_CONH_2_ solution and (D) 1000 mmol∙kg^-1^ NH_2_CONH_2_ solution at 298.15 K.

Attension tensiometer (Sigma 701, Germany) was utilized to determine surface tension (*γ*) by the ring detachment process. The complete procedure has given previously in many research papers [31]. The *γ* of individual compounds along with their mixtures in various studied ratios were assessed by adding of stock solution in distilled water (without any additive)/in the attendance of electrolyte (50 mmol.kg^–1^ NaCl)/urea (500 mmol.kg^–1^ along with 1000 mmol.kg^–1^) at 298.15 K. These processes were repeated until obtaining a value of *γ* that was constant. The *cmc* values of studied compounds along with their mixtures were attained from the intersect spot in a graph of the *γ* versus logarithm of compound concentration and accurateness of the *γ* measurement used was found to be near ±0.2 mN m^–1^. The inaccuracy in temperature is diminished to 0.2 K along with the relative uncertainties limits in *cmc* were achieved about 3%. Typical classical plots of variation of *γ* against concentration of individual and mixed amphiphiles are displayed in Figs 3 and 4.

#### Fluorescence study

For the purpose of aggregation number (*N*_agg_) determination, the fluorescence measurements for the individual drug, green gemini surfactant plus their solution mixtures in absence along with the presence of electrolyte/urea were performed by means of a F-7500 fluorescence spectrophotometer (Hitachi, Japan) via a 10 mm path length quartz cuvette at constant temperature (298.15 K). The spectra were measured amongst 350 and 450 nm using 335 nm excitation wavelengths. In favor of the evaluation of aggregation number (*N*_agg_), the concentration of the individual drug, green gemini surfactant in addition to their solution mixtures were formed higher than their relevant *cmc* value. The relative uncertainties for *N*_agg_ were calculated at approximately 4%. In this study, pyrene (PR) is utilized as a probe while cetylpyridinium chloride (CC) is used as a quencher.

#### FT-IR study

The FT-IR spectrum was recorded within the region between 4000 and 400 cm^–1^ and a selected portion was provided in the article. A Thermo Scientific NICOLET iS50 FT-IR spectrometer (Thermo Scientific, Madison, USA) was employed for recording the spectra. The samples of pure gemini (16-E2-16), drug (AMH) along with their mixtures in equal ratio were prepared in distilled water. The spectrum of H2O was taken off from the prepared associate system.

## Results and discussion

### cmc and cmc^id^ in absence or attendance of additive

Surface tension evaluation is an excellent technique to disclose the adsorption conduct of the molecules at a surface. In aqueous, solution the micellization phenomenon primarily relies upon the electrostatic interactions amongst the head groups of amphiphile along with hydrophobic interactions amongst the hydrocarbon chain of amphiphile monomers [5]. The interaction between the antidepressant drug amitriptyline hydrochloride (AMH) and the green gemini surfactant, 16-E2-16 was evaluated through tensiometry and fluorometry methods in water/NaCl/urea solutions. Latest analysis has revealed the utilization of gemini surfactants as a result of their advanced properties containing extremely small *cmc* value compared with the traditional ones because it holds two head groups as well as two hydrocarbon chains in a single monomer that increases the hydrophobicity too much [32]. In the current study, *cmc*_s_ of the individual in addition to mixed amphiphile systems were assessed by the surface tension methods in the absence/occurrence of salt/urea. Tensiometry can disclose the air–solution interface surface phenomena of the system concerning the processes in the bulk and is employed to examine the interactions amongst ingredients at the interfacial surface. The variation of surface tension (*γ*) of the drug, gemini as well as their mixtures in the absence/existence of 50 mmol.kg^–1^ NaCl along with 500 mmol.kg^–1^ and 1000 mmol.kg^–1^ urea is exposed in Figs 3 and 4. As shown in Figs 3 and 4 the predictable conduct of a sudden change of slope at the critical micelle concentration (*cmc*) and it is significant to point out that in the current study lack of minima is obtained near the breakpoint, confirming the high purities of employed chemicals [5]. The value of *cmc* of pure AMH, 16-E2-16 together with their mixed systems in various ratios (0.1 16-E2-16 + 0.9 AMH, 0.3 16-E2-16 + 0.7 AMH, 0.5 16-E2-16 + 0.5 AMH, 0.7 16-E2-16 + 0.3 AMH and 0.9 16-E2-16 + 0.1 AMH) in the absence/presence of electrolyte/urea is given in Table 2. Different ratios mean ratios in different mole fractions (*α*) of both employed constituents (AMH and 16-E2-16). Here, we have considered that *α*_1_ is the mole fraction of 16-E2-16 (first constituent) and *α*_2_ is the mole fraction of AMH, i.e., second constituent (*α*_1_ + *α*_2_ = 1).

**Table 2:** Physicochemical parameters for AMH-16-E2-16 mixtures in different media at temperature T = 298.15 K and pressure p = 0.1 MPa^a^.

*α*_1_	*cmc* (mmol·kg^-1^)	*cmc*^id^ (mmol·kg^-1^)	*X*_1_^Rub^	*X*_1_^id^	*β*^Rub^	*f*_1_^Rub^	*f*_2_^Rub^	ln(*cmc*_1_/*cmc*_2_)
Aqueous system								
0	32.36							
0.1	0.10	0.134	0.8461	0.9962	-5.61	0.8754	0.0179	
0.3	0.037	0.045	0.8886	0.9990	-6.26	0.9253	0.0072	
0.5	0.020	0.027	0.8633	0.9996	-8.19	0.8580	0.0022	-7.78
0.7	0.013	0.019	0.8480	0.9998	-9.94	0.7949	0.0008	
0.9	0.010	0.015	0.8540	0.9999	-11.62	0.7806	0.0002	
1	1.35x10^-2^							
50 mmol∙kg^-1^ NaCl								
0	29.75							
0.1	0.089	0.102	0.9063	0.9969	-4.32	0.9627	0.0287	
0.3	0.03	0.034	0.9173	0.9992	-5.68	0.9619	0.0084	
0.5	0.016	0.020	0.8796	0.9996	-7.91	0.8916	0.0022	-7.98
0.7	0.011	0.015	0.874	0.9998	-9.23	0.8637	0.0009	
0.9	0.0085	0.011	0.8789	0.9999	-10.81	0.8534	0.0002	
1	1.02x10^-2^							
500 mmol∙kg^-1^ urea								
0	36.31							
0.1	0.13	0.147	0.9087	0.9963	-4.07	0.9666	0.0346	
0.3	0.043	0.049	0.9112	0.9991	-5.63	0.9565	0.0093	
0.5	0.025	0.029	0.9024	0.9996	-6.95	0.9359	0.0034	-7.81
0.7	0.016	0.021	0.8737	0.9998	-9.01	0.8661	0.0010	
0.9	0.012	0.016	0.872	0.9999	-10.87	0.8368	0.0003	
1	1.48x10^-2^							
1000 mmol∙kg^-1^ urea								
0	39.80							
0.1	0.16	0.172	0.9369	0.9961	-3.26	0.9871	0.0573	
0.3	0.054	0.058	0.9479	0.9989	-4.49	0.9878	0.0176	
0.5	0.03	0.035	0.9121	0.9996	-6.56	0.9506	0.0043	-7.74
0.7	0.02	0.025	0.8917	0.9998	-8.27	0.9075	0.0014	
0.9	0.015	0.019	0.8875	0.9999	-10.16	0.8792	0.0003	
1	1.73x10^-2^							

^a^Standard uncertainties (*u*) are *u*(*T*) = 0.20 K, *u*(*NaCl*) = 1 mmol∙kg^-1^, *u*(*urea*) = 2 mmol∙kg^-1^ and *u*(*p*) = 5 kPa (level of confidence = 0.68). Relative standard uncertainties (*u*_*r*_) are *u*_*r*_(*cmc/cmc*^id^) = ±3%, *u*_*r*_(*X*_1_^Rub^/*X*_1_^id^) = ±3%, *u*_*r*_(*β*^Rub^) = ±3%, and *u*_*r*_(*f*_1_^Rub^/*f*_2_^Rub^) = ±4%.

In aqueous medium, the *cmc* value of individual drug AMH at 298.15 K was 32.36 mmol∙kg^–1^ showing the evaluate value is near to prior accounted value [1,33,34]. However, the *cmc* value for the individual gemini (16-E2-16) was obtained to be 1.35 x 10^−2^ mmol∙kg^–1^ that was too in fine agreement through the previously accounted value [29,30]. Here is notable that the value of *cmc* of 16-E2-16 is found to be a large amount lower than amitriptyline hydrochloride (AMH). The hydrophobic part of the drug molecule is tiny plus rigid as is apparent from Fig 1 and as a result forms associated structure at higher concentration. The very low *cmc* of 16-E2-16 is due to the two cationic head groups that are connected by a spacer; therefore the electrostatic repulsion between headgroups was hindered. The 16-E2-16 can pack strongly at the interfacial surface, along with their surface activities being significantly increased.

The *cmc* value of singular drug (AMH), gemini surfactant (16-E2-16) plus their mixtures reduces in the attendance of salt but in the attendance of NH_2_CONH_2_ (500 and 1000 mmol.kg^–1^) their *cmc* values were increased (Table 2). The *cmc* values were lowered by the majority of electrolytes considered to date, signifying inorganic salt anions directs to the shrinking of the width along with the potential of the electric double layer at the interfacial surface.^5^ In our case also *cmc* of studied systems of amphiphiles decrease largely through the addition of NaCl (inorganic salt) (Table 2) [5,35]. The reason can be clarified by the interactions between electrolyte and amphiphile. In the case of ionic amphiphiles, the counterions effect chiefly in the reduction of electrostatic repulsion involving their ionic headgroups, and in that way diminishing the effectual vicinity per head group supporting the formation of micelles [5,35]. The repulsion amongst head groups of employed ingredients is the main ruler characteristics for postponement of association [5,35].

The *cmc* values of pure and mixture of amphiphiles were increased by means of added urea. Usually, the actions of micelles in the presence of urea could be clarified in language of decrease of hydrophobic interactions through urea, which works as a water structure breaker [36]. Urea is liable to stabilize the amphiphiles monomer because urea increases the solubility of hydrocarbons in aqueous solution. In addition, urea may increase the repulsive force between the polar amphiphiles monomer head group of each amphiphile at the micellar surface. As a result, the start of association of monomers of drug (AMH) and surfactant (16-E2-16) along with mixtures in various ratio is delayed in the existence of NH_2_CONH_2_ (500 mmol.kg^–1^), contributing in boosting of *cmc* of the solutions and an additional rise in *cmc* value occurs through more increases in the concentration of urea (1000 mmol.kg^–1^).

**Fig 5:**
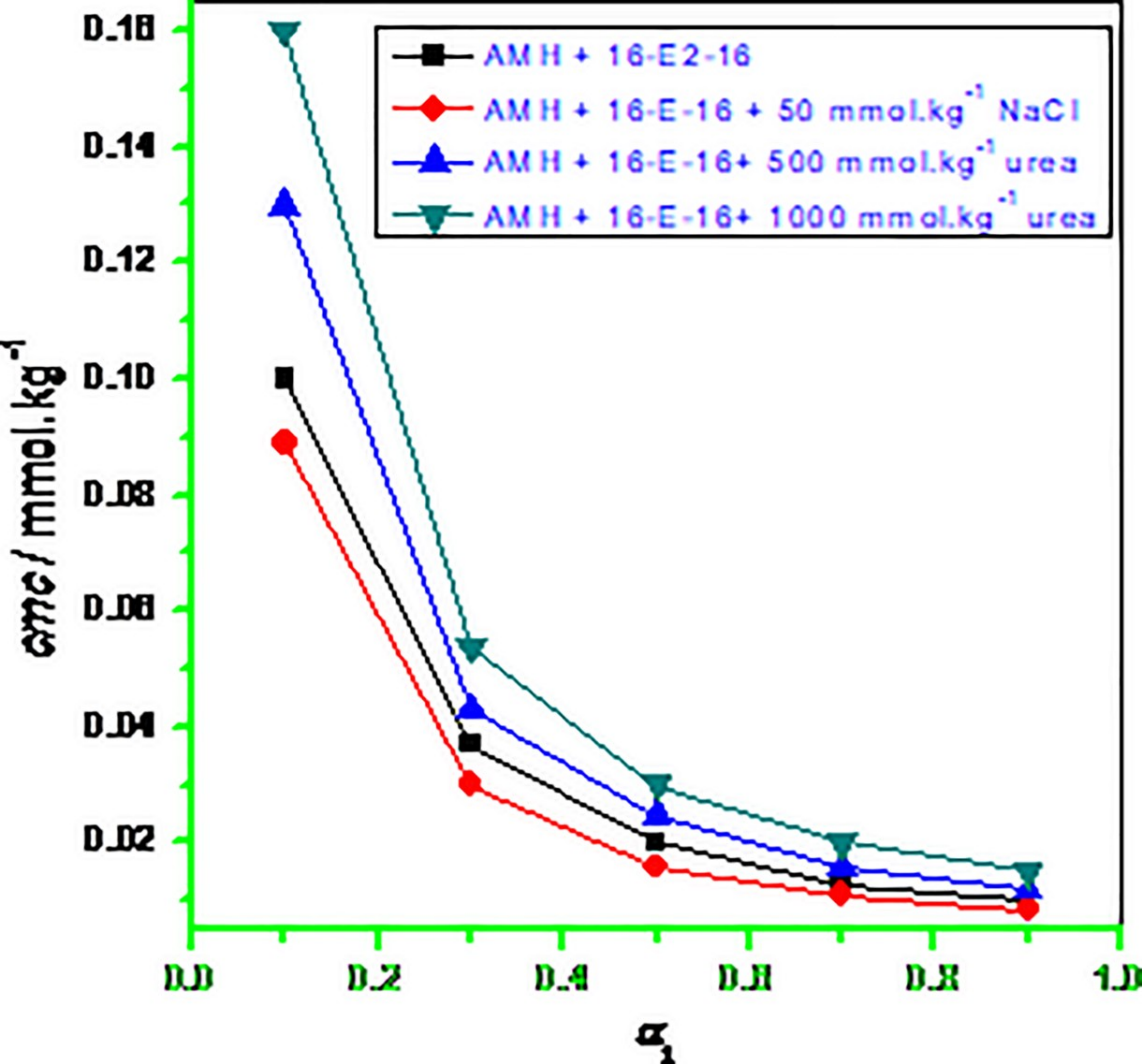
Variation of *cmc*/*cmc*^id^ of AMH-16-E2-16 mixture versus mole fraction (*α*_1_) of 16-E2-16.

In the mixtu**r**es of constituents, subsequent to an addition of certain concentration of prepared solution into aqueous or in presence of salt/urea system the continuing reduction of surface tension showed the positive adsorption of the AMH, 16-E2-16 as well as AMH–16-E2-16 mixtures at the solution interface. In the mixtures of AMH and 16-E2-16, as the *α*_1_ (mole fraction of gemini) rises in the solution, lowering of *cmc* value arises in all cases suggesting fine interaction between the involving ingredients (Table 2 along with Fig 5). Taking into consideration, as the *cmc* value of gemini surfactant is found to very low in comparison with the *cmc* of AMH, as a result, 16-E2-16 will produce micelles instantly, along with the AMH monomers only intercalate into gemini (16-E2-16) micelles signifies that mixed micelles of drug and gemini are a rich source in 16-E2-16 ingredients (Table 2 along with Fig 5).

To show the ideality in case of mixed micelle formation, one can employ Clint’s model [37]. This theory narrates the theoretical *cmc* (*cmc*^id^) of the ideal mixed system to the experimentally evaluated *cmc* values (*cmc*_1_ as well as *cmc*_2_) of the individual constituents and they believe that both constituents are noninteracting. The *cmc*^id^ value for a mixed system of two constituents is calculated by employing Eq (1) [37].

$$\frac{1}{cmc^{\mathrm{id}}}=\frac{\alpha_{1}}{cmc_{1}}+\frac{\alpha_{2}}{cmc_{2}}$$ (1)

In Eq (1), *α*_1_ is the mole fraction of ingredient first i.e., for 16-E2-16, *α*_2_ is the mole fraction of second ingredient i.e., AMH, *cmc*_1_ is the *cmc* of first constituent (16-E2-16) and *cmc*_2_ is the *cmc* of the drug (second constituent). The dissimilarity amongst the experimental *cmc* and *cmc*^id^ values provides an extent of nonideality in a mixture of the solution. If evaluated values of *cmc* were obtained more than *cmc*^id^, this means the system shows synergism (attractive interactions) in spite of this fact that if *cmc* values were found to be less than *cmc*^id^ then the system shows antagonism (repulsive interactions) behavior.

To see the type of interactions between the ingredients along with the deviation from ideality, all *cmc* values of mixtures (experimental as well as calculated) at various *α*_1_ of gemini are given in Table 2. Obtained data reveals that at all mole fractions of gemini (*α*_1_), the experimental *cmc* values were achieved below as compared to computed (calculated) values of *cmc* (*cmc*^id^ (ideal *cmc*)) (Table 2) [38]. So, the attained results identify nonideal behavior implies a negative deviation with ideality, showing the striking interactions between the constituents [39]. In existence of salt in mixed systems (drug–16-E2-16), the divergence from ideality was attained more as compared with their absence, while the deviation from ideality was found to be low in existence of urea. The deviations from ideality were further decreased through the boost of the amount of urea (from 500 to 1000 mmol∙kg^–1^ NH_2_CONH_2_) in solution mixtures. The considerable lowering of *cmc* is because of the increased hydrophobicity (intercalation of AMH monomers into the micelles of 16-E2-16 screens the repulsive interaction), in addition hydrophobicity also increases.

#### Interaction between AMH and 16-E2-16

Rubingh projected the familiar regular solution theory (RST) to suitably foresee the *cmc* of every mixed system of two amphiphiles based on the *cmc* values of the pure amphiphiles [40]. RST has been verified as a strong basis for estimating the nonideality of binary mixtures, principally as it presented a relatively simple however valuable quantitative tool interaction parameter (*β*^Rub^) to explain familiar amphiphile synergism along with antagonistic happening. The following equation was proposed by Rubingh to evaluate the micellar composition for nonideal solution mixtures [40]:

$$\frac{{\left(X_{1}^{\mathrm{Rub}}\right)}^{2}\ln\left(\alpha_{1}cmc/X_{1}^{\mathrm{Rub}}cmc_{1}\right)}{{\left(1-X_{1}^{\mathrm{Rub}}\right)}^{2}\ln\left[\left(1-\alpha_{1}\right)cmc/\left(1-X_{1}^{\mathrm{Rub}}\right)cmc_{2}\right]}=1$$ (2)

In the Eq (2) $X_{1}^{Rub}$ is the micellar composition (micellar mole fraction) of ingredient 1 (16-E2-16) in the mixed micelle of AMH–16-E2-16 mixtures. Eq 2 needs to be solved by iteration and subsequently the interaction parameter (*β*^Rub^) amongst two constituents in a mixed micelle can be attained suitably by employing Eq (3):

$$\beta^{\mathrm{Rub}}=\frac{\ln\left(\alpha_{1}cmc/X_{1}^{\mathrm{Rub}}cmc_{1}\right)}{{\left(1-X_{1}^{\mathrm{Rub}}\right)}^{2}}$$ (3)

In addition, in an ideal solution, the *α*_1_ of constituent 1 in mixed micelle can be computed by means of pseudophase separation model (Eq (4)) [41].

$$X_{1}^{\mathrm{id}}=\frac{\alpha_{1}cmc_{2}}{\alpha_{1}cmc_{2}+\alpha_{2}cmc_{1}}$$ (4)

The evaluated values of $X_{1}^{Rub},\;X_{1}^{id}$ as well as *β*^Rub^ are shown in Table 2. The $X_{1}^{Rub}$ value for 16-E2-16 is achieved higher as compared to the used *α*_1_ of 16-E2-16 excluding at *α*_1_ = 0.9. This proposes that a higher quantity of 16-E2-16 were attained in the mixed micelles and a lower fraction of drug there. The $X_{1}^{id}$ values increases through the rise in *α*_1_ of 16-E2-16 and their values were always above the *α*_1_ values means their values of course are higher than $X_{1}^{Rub}$ showing that mixed micelles enclose fewer 16-E2-16 as expected from ideal behavior. The $X_{1}^{Rub}$ values are found to be more in the occurrence of electrolyte viewing that the fraction of 16-E2-16 rises in mixed micelles in comparison to their absence. As NaCl dwindling of repulsive interactions amongst head groups, therefore, $X_{1}^{Rub}$ values enhances, that's why lessening in *cmc* of AMH–16-E2-16 mixture is also more in the presence of salt. Accordingly, we firmly obtained that the AMH–16-E2-16 mixture micelles formation takes place easily in salt solution as compare with aqueous systems. In the existence of NH_2_CONH_2_ in solution, the $X_{1}^{Rub}$ does not show a particular pattern by means of *α*_1_ (Table 2).

The interaction parameter represented via *β*^Rub^, provides information regarding the attractive interaction amongst involving ingredients. The analyzed values of *β*^Rub^ in all media are recorded in Table 2. The *β*^Rub^ values, possibly will be positive called antagonistic interaction or negative called synergistic interaction and in some cases zero means zero interaction between constituents) [5]. In addition, a negative *β*^Rub^ value denotes that the attractions between the constituents are above the pure constituents. In the current system all *β*^Rub^ values emerges negative in every binary mixture, alternatively their negative values increases as the mole fraction of 16-E2-16 increases. Negative *β*^Rub^ values were usually accountable for encouraging interactions between constituents, on the other hand positive values signify adverse interactions [5,42]. Our *β*^Rub^ value ranges from –11 to –3 (Table 2) showing well built attractive interactions among the ingredients. In any system synergism interaction during the progression happens as the *cmc* of the mixed systems are lesser than both individual constituents (*cmc* < *cmc*_1_, *cmc*_2_) along with it is proved as the subsequent two states are satisfied [2]: (a) *β*^Rub^ is negative plus (b) |*β*^Rub^|>|ln(*cmc*_1_/*cmc*_2_)|. Herein, from the above given two circumstances only the first condition is satisfied at all mole fractions but only at higher mole fraction is the second condition followed. For this reason, it is right to take up the word attractive interaction between the studied constituent at inferior *α*_1_ while at higher *α*_1_ synergism interaction between constituents occurs. In the existence of 500 mmol.kg^–1^ NH_2_CONH_2_, the value *β*^Rub^ was found to be less in comparison with the aqueous system, signifying that the interaction among components diminishes (Table 2). The decrease in the value of *β*^Rub^ happens because NH_2_CONH_2_ unites freely by the hydrophobic portion of solute moreover lowers the hydrophobicity causing the rise in the *cmc* value together with the reduction of the *β*^Rub^ values (Table 2). The *β*^Rub^ values were further reduced through an enhance in the concentration of NH_2_CONH_2_.

Activity coefficients ($f_{1}^{Rub}$ as well as $f_{2}^{Rub}$) of both amphiphiles within the mixed micelles are evaluated by employing the values of $X_{1}^{Rub}$ and *β*^Rub^ using Eqs (5) and (6):

$$f_{1}^{\mathrm{Rub}}=\exp\left[\beta^{\mathrm{Rub}}{\left(1-X_{1}^{\mathrm{Rub}}\right)}^{2}\right]$$ (5)

$$f_{2}^{\mathrm{Rub}}=\exp\left[\beta^{\mathrm{Rub}}{\left(X_{1}^{\mathrm{Rub}}\right)}^{2}\right]$$ (6)

As exposed in Table 2, the values of $f_{1}^{Rub}$ as well as $f_{2}^{Rub}$ in aqueous as well as other media at the entire studied *α*_1_ are less than unity, demonstrating the existence of mixed micelles in the solution means attractive interactions as well as nonideal behavior of the mixed systems. The above results also show that the state of drug and surfactant mixtures in all media is far from the ideal one. It is also found that the values of $f_{1}^{Rub}$ are more than the $f_{2}^{Rub}$ which points out a larger part of 16-E2-16 surfactant in the mixed micelle.

### Interfacial properties of AMH–16-E2-16 mixture

In water and in the presence of salt/urea the maximum excess surface concentration (*Γ*_max_) as well as the area taken by a single amphiphile monomer at the interfacial surface (*A*_min_) was achieved by employing the Gibbs adsorption equation [43,44]. A lower *A*_min_ or a larger *Γ*_max_ indicates a thicker packing of amphiphile monomers in the system. We can compute the maximum excess surface concentration (*Γ*_max_) by means of Eq (7) [43,44]:

$$\Gamma_{\max}=-\frac{1}{2.303nRT}\left[\frac{\partial \gamma }{\partial \log\left(m\right)}\right]\;\left(\mathrm{mol}\;m^{-2}\right)$$ (7)

In Eq (7), *m* is the concentration of amphiphile, *R* and *T* have standard meaning, ∂*γ*/∂1og(*m*) is the slope observed between plot of *γ* and log of the concentration (*m*), and *n* is the total count of species constituting the amphiphile monomers absorbed at the interfacial surface [5]. The *n* is employed as 2 for individual AMH and for gemini (16-E2-16) *n* is equal to 3. However, in the case of a mixture of solutions the value of *n* was estimated by the relationship $n={n_{1}X}_{1}^{\sigma }+{n_{2}(1-X}_{1}^{\sigma })$ in water together with the presence of additive [45]. $X_{1}^{\sigma }$ is the molar composition in the mixed interface. The slope at any chosen concentration of the γ against log[*m*] graph is employed to evaluate the *Γ*_max_ value.

The minimum area per amphiphile monomer (*A*_min_) at the air-solution interface was assessed according to the subsequent Eq (8) [45,46]:

$$A_{min}=\frac{10^{20}}{N_{A}\Gamma_{\max}}\;\left({Å}^{2}\right)$$ (8)

where *N*_A_ is the Avogadro constant. The value of *A*_*min*_ reveals the compactness of the amphiphile at the air-solution interface. The values of *Γ*_max_ and *A*_*min*_ for the entire systems in H_2_O along with the presence of additives are shown in Table 3. The value of *Γ*_max_ as well as *A*_*min*_ in the case of pure 16-E2-16 was obtained in fine conformity with the previously stated value [47]. The *Γ*_max_ value is found to be more for AMH in comparison with 16-E2-16, whereas the values of *A*_*min*_ have the opposite trend. In the case of 16-E2-16 in aqueous solution, the Coulombic repulsion involved both likewise charged head groups’ outcomes in a critical distance between both heads. As a result, the spacer of small length residue was entirely extended and occupies additional space. For this reason, *A*_*min*_ increases by means of the length of the small spacer.

**Table 3:** Surface parameters for AMH-16-E2-16 mixtures in different media at temperature *T* = 298.15 K and pressure *p* = 0.1 MPa^a^.

*α*_1_	*X*_1_^σ^	*β*^σ^	*f*_1_^σ^	*f*_2_^σ^	*Γ*_max_ 10^7^ (mol m^-2^)	*A*_min._/ *A*^id^ (Ǻ^2^)	*γ*_cmc_	*π*_*cmc*_ (mN m^-1^)	*pC*_20_	ln(*conc*_1_/*conc*_2_)
Aqueous system										
0					20.13	82.49	42.48	28.52	1.87	
0.1	0.9789	-2.60	0.9988	0.0826	14.25	116.52/138.32	38.13	32.87	4.53	
0.3	0.9435	-5.50	0.9826	0.0075	11.65	142.56/136.30	38.07	32.93	5.08	
0.5	0.8789	-8.63	0.8811	0.0013	10.95	151.60/132.61	38.94	32.06	5.38	-8.52
0.7	0.8510	-10.87	0.7855	0.0004	11.38	145.84/131.02	37.44	33.56	5.59	
0.9	0.8861	-11.22	0.8645	0.0002	12.89	128.73/133.02	37.92	33.08	5.64	
1					11.90	139.52	36.98	34.02	5.57	
50 mmol∙kg^-1^ NaCl										
0					20.37	81.49	43.04	27.96	1.86	
0.1	0.8624	-6.77	0.8796	0.0065	12.57	132.08/121.17	34.71	36.29	4.86	
0.3	0.8819	-7.97	0.8947	0.0020	11.90	139.51/122.07	34.92	36.08	5.32	
0.5	0.8501	-10.30	0.7934	0.0006	12.81	129.56/120.60	34.40	36.60	5.61	-8.93
0.7	0.9249	-8.57	0.9528	0.0006	13.75	120.79/124.04	34.76	36.24	5.64	
0.9	0.9560	-8.87	0.9829	0.0003	16.16	102.77/125.48	34.75	36.25	5.65	
1					13.02	127.50	34.36	36.64	5.74	
500 mmol∙kg^-1^ urea										
0					18.57	89.41	42.43	28.57	1.84	
0.1	0.9311	-4.37	0.9795	0.0228	13.27	125.08/125.03	35.88	35.12	4.59	
0.3	0.8732	-7.73	0.8831	0.0028	11.61	142.96/122.82	35.94	35.06	5.14	
0.5	0.9073	-7.71	0.9359	0.0018	14.30	116.06/124.12	33.79	37.21	5.32	-8.54
0.7	0.9239	-8.16	0.9538	0.0009	14.97	110.89/124.76	34.05	36.95	5.45	
0.9	0.9482	-8.80	0.9766	0.0004	17.12	96.98/125.69	34.22	36.78	5.49	
1					13.0	127.67	35.57	35.43	5.55	
1000 mmol∙kg^-1^ urea										
0					14.06	118.05	44.09	26.91	1.83	
0.1	0.8384	-7.36	0.8251	0.0057	10.55	157.34/147.38	33.56	37.44	4.82	
0.3	0.8748	-8.05	0.8814	0.0021	10.87	152.74/148.66	33.89	37.11	5.25	
0.5	0.9663	-5.86	0.9933	0.0042	12.80	129.74/151.86	34.26	36.74	5.26	-8.82
0.7	0.9082	-9.04	0.9266	0.0006	11.48	144.62/149.83	34.13	36.87	5.58	
0.9	0.9383	-9.48	0.9645	0.0002	11.92	139.25/150.88	34.49	36.51	5.55	
1					10.85	153.04	34.38	36.62	5.66	

^a^Standard uncertainties (*u*) are *u*(*T*) = 0.20 K, *u*(*NaCl*) = 1 mmol∙kg^-1^, *u*(*urea*) = 2 mmol∙kg^-1^ and *u*(*p*) = 5 kPa (level of confidence = 0.68). Relative standard uncertainties (*u*_*r*_) are *u*_*r*_(*X*_*1*_^σ^) = ±2%, *u*_*r*_(*β*^σ^) = ±3%, *u*_*r*_(*f*_1_^σ^/*f*_2_^σ^) = ±4%, *u*_*r*_(*Γ*_max_) = ±5%, *u*_*r*_(*A*_min_/*A*^id^) = ±5%, *u*_*r*_(*π*_*cmc*_) = ±2%, *u*_*r*_(*pC*_20_) = ±3% and *u*_*r*_(*γ*_*cmc*_) = ±2%.

In existence of electrolyte the *Γ*_max_ value of pure constituents is increased indicating the *A*_*min*_ value is decreased than their corresponding salt-free systems as shown in Table 3. This phenomenon indicates that the effectiveness of the amphiphile molecules to occupy the interfacial surface is increased in the presence of NaCl, indicating the compaction of the 16-E2-16 and drug (AMH) monomers at the interfacial surface. In the case of mixtures of AMH and 16-E2-16 in the occurrence of salt similar behavior was obtained except for 0.1 *α*_1_ of gemini. However, in presence of NH_2_CONH_2_, the *Γ*_max_ value does not show any regular trend in all cases but overall systems show that their value reduces along with their value additional decreases via rise in amount of urea in the system. In the presence of urea, the H_2_O molecules around the hydrocarbon parts are substituted by urea, which increases the hydrophilicity of studied pure as well as mixed monomer micelles along with an increase in solubility of constituents. The happening of the above phenomena reduces the adsorption of constituents at the interfacial surface, accordingly *Γ*_max_ values decrease. Another reason is that owing to the existence of NH_2_CONH_2_ the increase in repulsive interaction expands the head groups of constituents at interfacial surface, in that way the decrease in the *Γ*_max_ value or increase in the *A*_*min*_ value occurs. By the increase in *α*_*1*_ of 16-E2-16 in the solution mixture the obtained value of *Γ*_max_ or *A*_*min*_ does not show any particular or regular trend in water as well as in the presence of urea/salt.

At ideal state minimum surface area (*A*^id^) per monomer has been analyzed through Eq (9):

$$A^{\mathrm{id}}=X_{1}^{\sigma }A_{1}+(1-X_{1}^{\sigma })A_{2}$$ (9)

In Eq (9), $X_{1}^{\sigma }$ is the molar composition of 16-E2-16 in mixed monolayer. *A*_1_ is the minimum area for each head group of 16-E2-16 as well as *A*_2_ is the minimum area for each head group of AMH. The experimental values of *A*_*min*_ of mixtures were achieved higher than the *A*^id^ along with *A*_*min*_ values mixtures were also more than the *A*_*min*_ value of pure constituents. This possibly as a result of the rigid in addition to large hydrophobic volumes of currently employed constituents (AMH and 16-E2-16) that generate steric hindrance.

A mixed monolayer is formed at interface of air and water surface prior to the formation of mixed micelles, by adsorption of amphiphiles. Similar to Rubingh’s [40] theory, Rosen gave a theory that is employed to elucidate the formation of a mixed monolayer of amphiphiles using Eq (10) [48]:

$$\frac{{\left(X_{1}^{\sigma }\right)}^{2}\ln\left(\alpha_{1}conc/X_{1}^{\sigma }\;conc_{1}\right)}{{\left(1-X_{1}^{\sigma }\right)}^{2}\ln\left[\left(1-\alpha_{1}\right)conc/\left(1-X_{1}^{\sigma }\right)conc_{2}\right]}=1$$ (10)

In the mixed monolayer, $X_{1}^{\sigma }$ specifies the mole fraction of 16-E2-16 at monolayer. The *conc* is the concentration of mixed monolayers (a mixture of AMH and 16-E2-16 in the absence and existence of NaCl and urea), *conc*_1_ is the concentration of 16-E2-16 and *conc*_2_ are the concentration of drug (AMH).

The interaction between components at air-solution interface, known as interaction parameter (*β*^σ^) for a mixed monolayer can be elucidated by employing Eq (11):

$$\beta^{\sigma }=\frac{\ln\left(\alpha_{1}conc/X_{1}^{\sigma }conc_{1}\right)}{{\left(1-X_{1}^{\sigma }\right)}^{2}}$$ (11)

The $X_{1}^{\sigma }$ along with *β*^σ^ values are shown in Table 3. For a binary mixed system, akin to *β*^Rub^, the *β*^σ^ is found to be zero for an ideal monolayer, while synergistic interaction was found if their values were obtained to be negative and antagonistic interaction between component were achieved for positive *β*^Rub^ value. It is clear from Table 3 that in our case negative *β*^σ^ values (akin to *β*^Rub^) were found, demonstrating the attractive interaction between the amphiphile molecules at the interfacial surface and their average value more or less also the same means interaction between component in mixed monolayer and in mixed micelles are nearly the same. For AMH–16-E2-16 mixture, $X_{1}^{\sigma }$ values were found to be comparable with the $X_{1}^{Rub}$ signifying that the mixed monolayer acquires nearly the same 16-E2-16 molecules as in the mixed micelles (Tables 2 and 3). The $X_{1}^{\sigma }$ values obtained are not demonstrating any definite or particular style through *α*_1_ of surfactant in the entire systems.

The synergism phenomenon in *γ* reduction efficiency is viewed, as the whole concentration of mixtures of the component in solution required decreasing, the *γ* of H_2_O toward a chosen value i.e., by 20 mNm^-1^ below to the singular ingredient. Furthermore this was validated after the subsequent both situations are satisfied [5]:

- *β*^*σ*^ is negative
- |*β*^*σ*^|>ln(*conc*_1_/*conc*_2_)

Although the estimated values of interaction parameters (*β*^σ^) at air–water interface are achieved negative as stated earlier but their negative values did not fulfill the second circumstances at every considered mole fraction of 16-E2-16, that is why the *β*^σ^ values of the studied system show synergism in *γ* reduction efficiency only at some mole fraction. Higher $\beta_{av}^{\sigma }$ (average) values for AMH–16-E2-16 mixed system in the presence of salt than for water or in the presence of urea confirm higher synergism (interaction) as well as higher nonideality at the interfacial surface as nearby is more electrostatic interaction amongst cationic head groups of mixed monolayer as NaCl diminishes the repulsion amongst the head groups [49].

The activity coefficients ($f_{1}^{\sigma }$ (16-E2-16) and $f_{2}^{\sigma }$ (AMH)) of both amphiphiles contained by the mixed monolayer are associated to the *β*^σ^ of the interface by the subsequent set of equations (Eqs (12) and (13)):

$$f_{1}^{\sigma }=\exp\left[\beta^{\sigma }{\left(1-X_{1}^{\sigma }\right)}^{2}\right]$$ (12)

$$f_{2}^{\sigma }=exp[{\beta^{\sigma }(X_{1}^{\sigma })}^{2}]$$ (13)

The evaluated value of both activity coefficients ($f_{1}^{\sigma }$ (16-E2-16) and $f_{2}^{\sigma }$ (AMH)) are shown in Table 3 along with other related parameters. In water along with the presence of additives, the values of $f_{1}^{\sigma }$ (16-E2-16) and $f_{2}^{\sigma }$ (AMH) are found to be less than unity, signifying the formation of a mixed monolayer, which suggests a nonideal behavior as well as positive interactions among the ingredients. Higher contribution of 16-E2-16 than the drug (AMH) constituents in the mixed monolayer, is pointed out by the larger values of $f_{1}^{\sigma }$ than $f_{2}^{\sigma }$. The existence of NH_2_CONH_2_ or salt in the solution not viewing any definite pattern in the values of $f_{1}^{\sigma }$ and $f_{2}^{\sigma }$.

The *γ* at *cmc* (*γ*_*cmc*_), effectiveness of *γ* reduction (*π*_*cmc*_), as well as adsorption efficiency (*pC*_20_) may possibly be employed to find out the surface activities of both considered ingredients along with mixtures. The π_*cmc*_ shows the maximum reduction of the *γ* and is identified by Eq (14) [5,50]:

$$\pi_{cmc}=\gamma_{o}–\gamma_{cmc}$$ (14)

In Eq 14, *γ*_0_ is the *γ* of H_2_O and *γ*_*cmc*_ is *γ* of the utilized amphiphiles at the *cmc*. The *γ*_*cmc*_ values for every studied system are depicted in Table 3. It is clear from the data that the *π*_cmc_ is found to be highest for AMH and lowest for 16-E-16, whereas for mixed systems the in-between values are found with the exception of 500 mmol.kg^–1^ urea but close to the pure 16-E2-16. The p*C*_20_ is normally employed to explain the efficiency of an amphiphile in lowering the *γ* of a solvent furthermore is computed by employing Eq (15) [5,51]:

$${pC}_{20}=-logC_{20}$$ (15)

In the above equation, *C*_20_ is the concentration needed to decline the *γ* of H_2_O by 20 mN·m^−1^. It specifies the adsorption efficiency of an individual amphiphile as well as their mixtures at the interfacial surface. The larger is the p*C*_20_ value, the less is the amount involved for lowering the *γ* value of solvent by 20 mN m^–1^. As shown in Table 3, the values of *pC*_20_ for 16-E2-16 were found to be much higher than the studied drug AMH in all different studied media. The outcome signified the superior surface activity of the gemini in comparison to the amphiphilic drug (AMH) [52]. Above obtained outcome is also proved via the very small *cmc* of 16-E2-16 as compared with AMH. The mixture of AMH–16-E2-16 possesses much more *pC*_20_ values as compared with pure AMH but near to *pC*_20_ value of gemini surfactant (Table 3). The value of *pC*_20_ increases by means of an increase in *α*_1_ of gemini surfactant means the surface activity of mixed system enhances through the rise in *α*_1_ of surfactant.

### Thermodynamics

Thermodynamic parameters of micellization for AMH, 16-E2-16 as well as their mixtures in various ratios were assessed for acquiring better knowledge regarding the association behavior of the amphiphile as well as intermolecular interactions present in the studied systems [5]. The Gibbs free energy ($\Delta G_{m}^{o}$) of association of amphiphiles mixtures could be attained through employing Eq (16) [53–55].

$$\Delta G_{m}^{o}=RT\;\ln X_{cmc}$$ (16)

**Fig 6:**
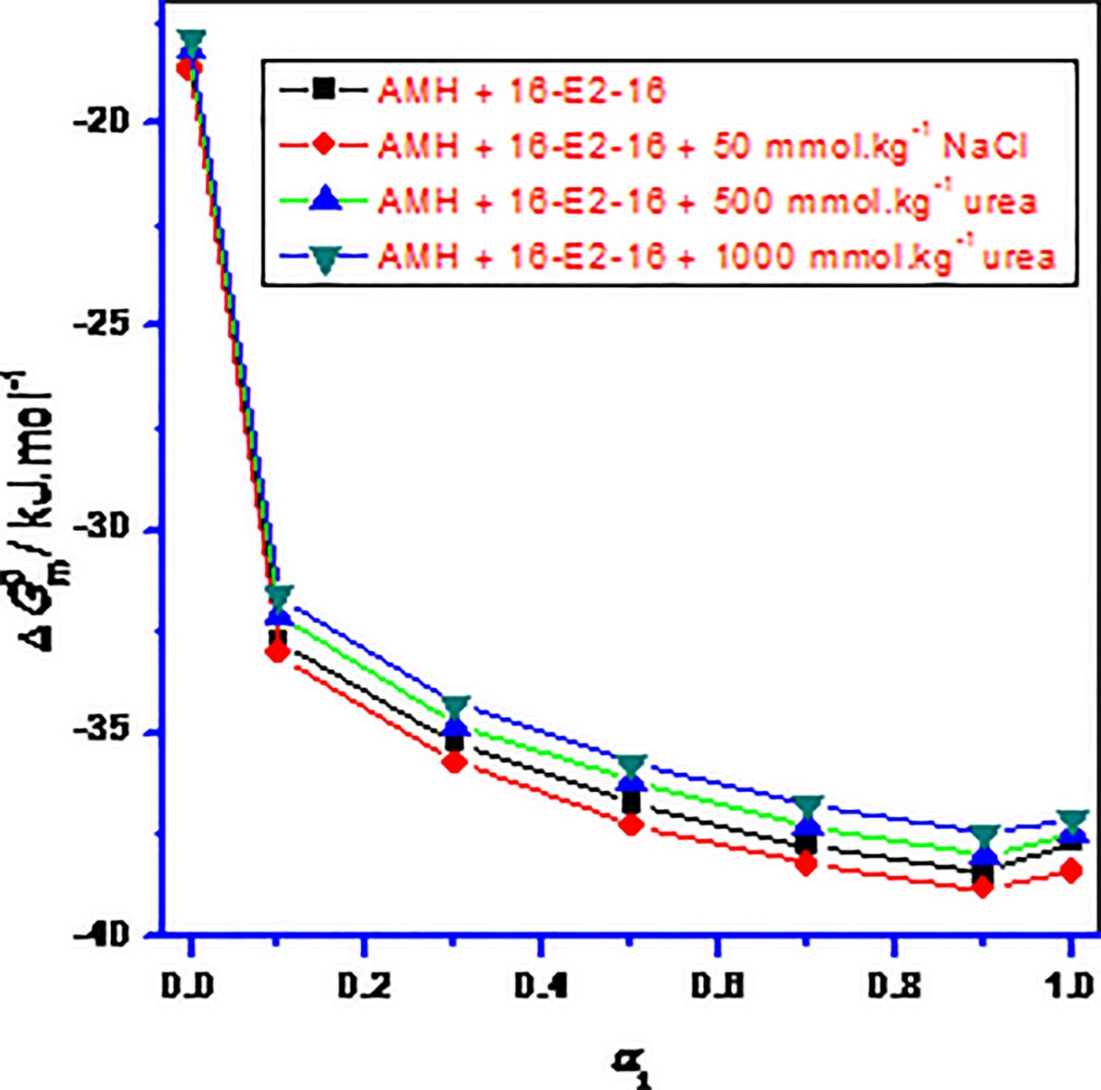
Variations of Δ*G*om versus mole fraction of 16-E2-16 (*α*_1_) in AMH-16-E2-16 mixed systems at temperature *T* = 298.15 K in different media at 298.15 K.

In Eq (16), *X*_*cmc*_ is *cmc* value in mole fraction. The pure plus their mixed studied systems the $\Delta G_{m}^{o}$ values were negative in all cases in different media (Fig 6) showing that the AMH, 16-E2-16 as well as AMH–16-E2-16 mixtures have a significantly spontaneous nature during micelle formation. The negative value of $\Delta G_{m}^{o}$ for all systems increases via increases in the *α*_1_ of 16-E2-16 demonstrating that their spontaneity further increases with increase in *α*_1_ of 16-E2-16 in the solution in three different media (Fig 6). The value of $\Delta G_{m}^{o}$ in case of pure AMH was acquired –18.34 kJ.mol^–1^, which is in very well consistency with the prior stated value along with being similar to other antidepressant drugs [56–58]. In addition to this, the value of $\Delta G_{m}^{o}$ for pure 16-E2-16 was also obtained in acceptable accord through the former disclosed value [17,27]. The $\Delta G_{m}^{o}$ value for the drug (AMH) was lower than the $\Delta G_{m}^{o}$ value for gemini (16-E2-16). This takes place by reason of small hydrophobicity of AMH as compared with 16-E2-16, which hinders aggregation phenomena to some extent. In the existence of electrolyte in a solution of individuals in addition to their mixtures, the values of $\Delta G_{m}^{o}$ were more negative as compared to aqueous system, illustrating that association initiates at lesser concentration seeing as energetic potency for aggregation is extensively increased (Fig 6). On the other hand, in the attendance of NH_2_CONH_2_ the $\Delta G_{m}^{o}$ value of solution was attained a lesser amount of negative signifying that aggregation was significantly reduced but the micellization phenomena are also thermodynamically spontaneous. Urea ruptures the H_2_O clusters near the hydrophobic fractions of AMH as well as 16-E2-16 as well as their mixtures and as a result it supports the hydration of the molecules that lessens the entropy expands throughout the period of aggregation. In view of that, the $\Delta G_{m}^{o}$ values become less negative for urea, in addition negative value reduces further by way of the boost in urea concentration.

The standard Gibbs energy of adsorption ($\Delta G_{ads}^{o}$) that is employed to compute the aggregation behavior of pure along with mixed system was estimated by means of the following equation [59,60]:

$$\Delta G_{ads}^{o}=\Delta G_{m}^{o}-\frac{\pi_{cmc}}{\Gamma_{max}}$$ (17)

In Eq (17), *π*_*cmc*_ is the effectiveness of surface tension reduction at the *cmc*, which is described as the surface enclosed through a monolayer of amphiphile at surface pressure equipped zero. The $\Delta G_{ads}^{o}$ values were found to be negative, suggesting the adsorption happening is spontaneous as given in Table 4 and their negative values were always obtained to be more than $\Delta G_{m}^{o}$. This phenomenon points out the adsorption at the interfacial surface is additional favorable as compared with the micelles formation in the bulk solution [5]. This obviously signifies that subsequent to micelle growth, work is needed to transport the residual amphiphile molecules from the surface to the direction of the micellar state in the pure component (AMH, 16-E2-16) plus their mixtures [61]. The value of $\Delta G_{ads}^{o}$ of AMH is fewer negative but more or less equal as compared with the mixture of constituents, signifying that the mixed systems are additional surface active as compared with singular AMH. The value of $\Delta G_{ads}^{o}$ does show any particular trend in the existence of NH_2_CONH_2_, both in the case of pure component in addition to their mixtures; however, in the presence of NaCl the $\Delta G_{ads}^{o}$ value for all systems are attained additional negative representing that adsorption is additionally spontaneous in the presence of electrolyte with few exceptions (Table 4).

**Table 4:** Thermodynamic parameters for AMH-16-E2-16 mixtures in different media at temperature *T* = 298.15 K and pressure *p* = 0.1 MPa^a^.

*α*_1_	Δ*G*^o^_ads_ (kJ mol^-1^)	*G*_min_ (kJ mol^-1^)	$\Delta G_{\mathrm{ex}}^{\sigma }$ (kJ mol^-1^)	$\Delta G_{\mathrm{ex}}^{m}$ (kJ mol^-1^)
Aqueous system				
0	-32.62	21.10		
0.1	-55.84	26.76	-0.13	-1.81
0.3	-63.51	32.69	-0.73	-1.53
0.5	-66.03	35.56	-2.28	-2.39
0.7	-67.30	32.89	-3.42	-3.17
0.9	-64.12	29.39	-2.81	-3.58
1	-66.32	31.08		
50 mmol∙kg^-1^ NaCl				
0	-32.38	21.13		
0.1	-61.93	27.61	-1.99	-0.91
0.3	-66.07	29.34	-2.06	-1.07
0.5	-65.87	26.84	-3.25	-2.08
0.7	-64.60	25.29	-1.48	-2.52
0.9	-61.31	21.51	-0.92	-2.85
1	-66.56	26.39		
500 mmol∙kg^-1^ urea				
0	-33.55	22.85		
0.1	-58.58	27.03	-0.69	-0.84
0.3	-65.05	30.95	-2.12	-1.13
0.5	-62.22	23.62	-1.61	-1.52
0.7	-61.99	22.74	-1.42	-2.46
0.9	-59.51	19.99	-1.07	-3.01
1	-64.75	27.35		
1000 mmol∙kg^-1^ urea				
0	-37.07	31.35		
0.1	-67.09	31.80	-2.47	-0.48
0.3	-68.44	31.18	-2.18	-0.55
0.5	-64.46	26.77	-0.47	-1.30
0.7	-68.87	29.73	-1.87	-1.98
0.9	-68.09	28.93	-1.36	-2.51
1	-70.87	31.69		

^a^Standard uncertainties (*u*) are *u*(*T*) = 0.20 K, *u*(*NaCl*) = 1 mmol∙kg^-1^, *u*(*urea*) = 2 mmol∙kg^-1^ and *u*(*p*) = 5 kPa (level of confidence = 0.68). Relative standard uncertainties (*u*_*r*_) are *u*_*r*_(Δ*G*^o^_m_) = ±3%, *u*_*r*_(Δ*G*^o^_ads_) = ±4%, *u*_*r*_(*G*_min_) = ±4% and $u_{r}({\Delta G}_{ex}^{m}/{\Delta G}_{ex}^{\sigma })$ = ±5%.

The excess free energy (${\Delta G}_{ex}^{m}$) values of mixed micellization along with mixed monolayer (${\Delta G}_{ex}^{\sigma }$) (between monomeric and micellar state) were estimated by means of the subsequent Eqs (18) and (19) [62–65]:

$${\Delta G}_{ex}^{m}=RT[X_{1}^{Rub}\;\ln f_{1}^{Rub}+(1-X_{1}^{Rub})lnf_{2}^{Rub}]$$ (18)

$${\Delta G}_{ex}^{\sigma }=RT[X_{1}^{\sigma }\;\ln f_{1}^{\sigma }+(1-X_{1}^{\sigma })lnf_{2}^{\sigma }]$$ (19)

The acquired ${\Delta G}_{ex}^{m}$ along with ${\Delta G}_{ex}^{\sigma }$ values have been given in Table 4. Table 4 shows that the values achieved for the mixed micelles as well as mixed monolayer were negative signifying the formation of mixed micelles along with mixed monolayer in the studied systems is more stable in contrast to singular amphiphile micelles as well as monolyer formation. The ${\Delta G}_{ex}^{\sigma }$ values were obtained as larger than ${\Delta G}_{ex}^{m}$ at maximum *α*_1_ of 16-E-16 demonstrating that the mixed monolayer is found to be additionally stable as compared with mixed micelles. This phenomenon also gets hold up from the values of minimum molar Gibbs free energy (*G*_*min*_).

The minimum molar Gibbs free energy (*G*_*min*_) of the particular interface attaining the maximum adsorption at *cmc*, is evaluated via employing the following equation [66]:

$$G_{min}=A_{min}\gamma_{cmc}N_{A}$$ (20)

*G*_*min*_ is the effort necessary to transport the amphiphilic monomers from the bulk phase to the interfacial surface of the amphiphile system. The poorer *G*_*min*_ value points out high thermodynamically stable surfaces. The experimental lower values of *G*_*min*_ verify the thermodynamic stable surface formation through entirely adsorbed amphiphilic molecules. With the change in mole fraction of 16-E2-16, the *G*_*min*_ value does not show any regular trend also in the presence of salts and urea (Table 4).

### Packing parameters of AMH–16-E2-16 mixed micelles

The values of *A*_min_ can be employed to compute the packing parameter (*P*), that illustrates the shape of the micelles. *P* can be evaluated by utilizing the following equation [67]:

$$P=\frac{V_{0}}{A_{min}l_{c}}$$ (21)

In Eq 21, *V*o points out the volume engaged via the hydrophobic portions in the micellar interior in present study along with *lc* symbolizing maximum effective chain length of the hydrophobic chain in the interior that is believed to be fluid and incompressible. The *V*o and *lc* values can be estimated through Tanford’s methods [68]:

$$V_{0}=[27.4+26.9\;(n_{c}-1)]\;({Å}^{3})$$ (22)

$$l_{c}=[1.54+1.26\;(n_{c}-1)]\;(Å)$$ (23)

In Eqs 22 and 23 *n*_c_ is the number of carbon (C) atoms in the saturated chain length. Solutions of every system of individuals along with their mixtures in the absence as well as the presence of additives were prepared above their *cmc* value and evaluated *P* (packing parameter) values are given in Table 5. Packing parameter (*P*) values can be employed to calculate the shape as well as kind of the micelle formed in different studied media. The sum total digits of C atoms is considered below one from the complete number of C atoms in the hydrocarbon chain (*n*_c_) of molecules. The opening carbon atom just behind the head group is very much solvated so probably measured as a piece of the head group [69].

**Table 5:** Packing parameter for mixed AMH-16-E2-16 systems in different media at temperature *T* = 298.15 K and pressure *p* = 0.1 MPa^a^.

*α*_1_	*V*_0_ (Å^3^)	*l*_c_ (Å)	*P*
Aqueous system			
0	1130.8	25.48	0.54
0.1	3713.2	85.96	0.37
0.3	3713.2	85.96	0.30
0.5	3713.2	85.96	0.28
0.7	3713.2	85.96	0.30
0.9	3713.2	85.96	0.34
1	2099.2	48.16	0.31
50 mmol∙kg^-1^ NaCl			
0	1130.8	25.48	0.54
0.1	3713.2	85.96	0.33
0.3	3713.2	85.96	0.31
0.5	3713.2	85.96	0.33
0.7	3713.2	85.96	0.36
0.9	3713.2	85.96	0.42
1	2099.2	48.16	0.34
500 mmol∙kg^-1^ urea			
0	1130.8	25.48	0.50
0.1	3713.2	85.96	0.35
0.3	3713.2	85.96	0.30
0.5	3713.2	85.96	0.37
0.7	3713.2	85.96	0.39
0.9	3713.2	85.96	0.45
1	2099.2	48.16	0.34
1000 mmol∙kg^-1^ urea			
0	1130.8	25.48	0.38
0.1	3713.2	85.96	0.27
0.3	3713.2	85.96	0.28
0.5	3713.2	85.96	0.33
0.7	3713.2	85.96	0.30
0.9	3713.2	85.96	0.31
1	2099.2	48.16	0.28

^a^Standard uncertainties (*u*) are *u*(*T*) = 0.20 K and *u*(*p*) = 5 kPa (level of confidence = 0.68). Relative standard uncertainties (*u*_*r*_) are *u*_*r*_(*V*_0_) = ±3%, *u*_*r*_(*l*_c_) = ±3% and *u*_*r*_(*P*) = ±4%.

Moreover, for achieved *P* values between 0 to 0.33; the probable formed associate’s arrangements are spherical or ellipsoidal in shape. On the other hand, if *P* values in between 0.33 to 0.5, then the probable shape of micelles is cylindrical or rod-shaped. However, if the value of *P* were obtained in between 0.5 and 1 then formed associate’s structure is vesicles or flexible bilayer in shape [5]. In the present case, the value of *P* for the drug (AMH) was found to above 0.5 except in the existence of 1000 mmol.kg^–1^ urea, viewing that the drug forms vesicles (Table 5). For gemini surfactant (16-E2-16) plus their mixtures with AMH were obtained in between 0.33 and 0.5 showing that micelles formed by stated systems are cylindrical or rod-shaped; however, at some mole fractions their values were found to be below 0.33 but close to 0.30 showing that formed associates structures called micelles are spherical or ellipsoidal in shape (Table 5) [70]. The 16-E2-16 has high repulsion on micellization. For that reason, tiny freely packed micelles are formed having small *P* value are obtained. Packing parameter (*P*) does not demonstrate any particular trend in presence of NaCl and urea.

### Fluorescence measurements

#### Aggregation number (*N*_*agg*_)

The measurement of fluorescence is frequently employed to examine the self-association of individual as well as mixed amphiphile systems in aqueous as well as nonaqueous systems by means of the emission of PR (pyrene) [26,71]. This technique means steady-state fluorescence quenching is a suitable process for a precise estimation of micellar association as well as aggregation number (*N*_agg_) [72]. The quenching of PR fluorescence via CC (quencher) is used to obtain the *N*_agg_ of micelles formation in case of pure amphiphile (AMH, 16-E2-16) plus their mixed systems in the entire ratios in all studied different media (aqueous/NaCl/NH_2_CONH_2_). For the determination of *N*_agg_ of the studied system following equation was employed [73,74]:

$$ln\left(\frac{I_{0}}{I_{1}}\right)=\frac{N_{agg}[Q]}{S_{T}-cmc}$$ (24)

**Fig 7:**
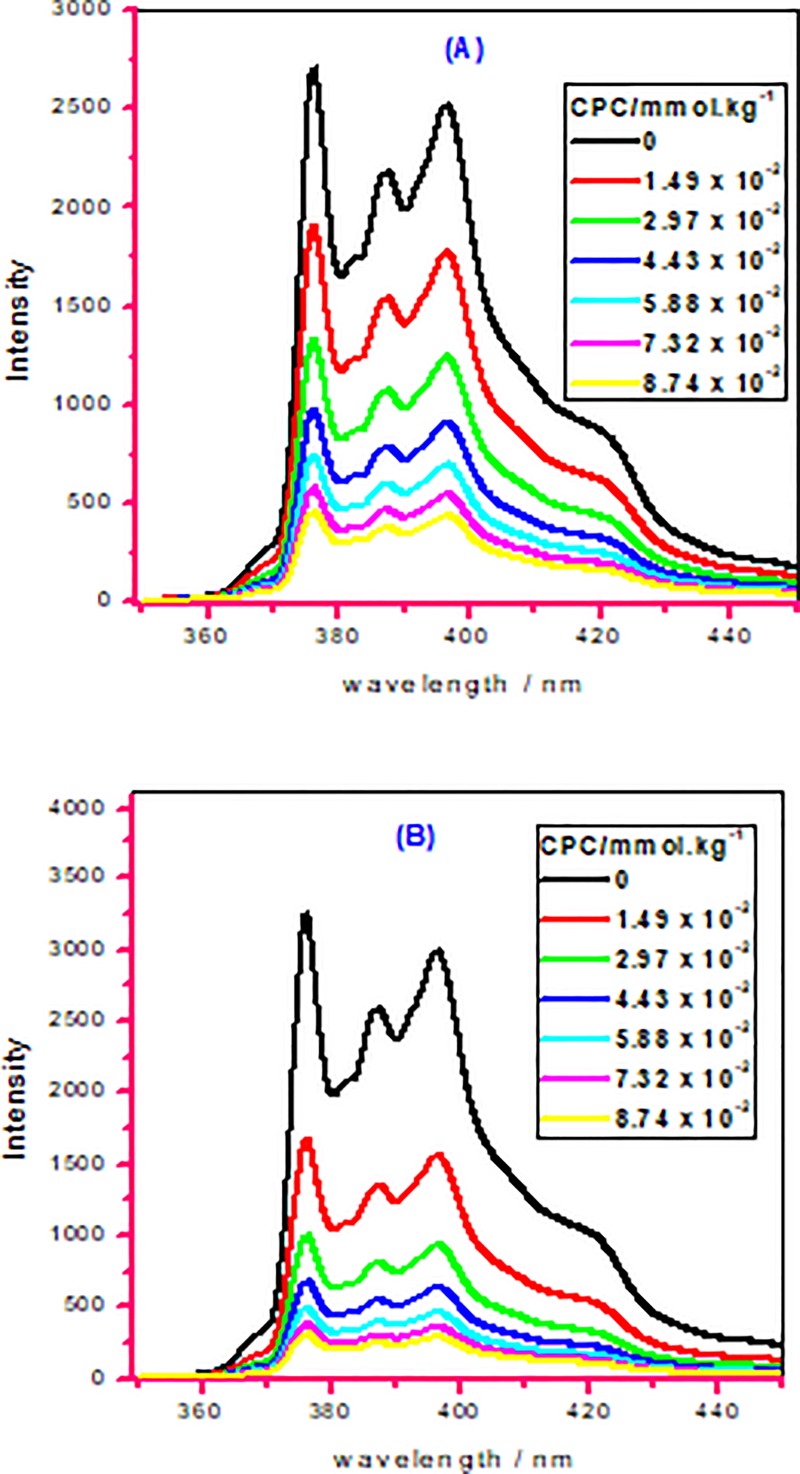
Fluorescence spectra of 10^−6^ M pyrene of (A) pure 16-E2-16, and (B) 16-E2-16 (0.4) + AMH (0.6) mixture at different quencher concentrations in aqueous micellar solution.

In Eq (24), *I*_0_ is fluorescence intensity in the absence of CC and *I*_1_ is also the same parameter but the presence of quencher (CC). [Q] and *S*_T_ are the employed concentration of quencher and the whole concentration of amphiphile (pure or mixed system), respectively. Fig 7 shows the change in fluorescence intensity of PR in the attendance of various concentrations of CC in micellar solution of (a) individual 16-E2-16 and (b) 16-E2-16 (0.4) + AMC (0.6) mixture. Every spectrum keeps five extremely separate emission bands beginning from smaller to upper wavelengths (370 to 400 nm). Eq 24 foresees a linear plot of ln(*I*_0_/*I*_1_) against [Q] having a slope come to *N*_agg_/([S_T_]–*cmc*) that grants the *N*_agg_ value. The obtained *N*_agg_ values of individual species (AMH (drug) and 16-E2-16 (gemini)) together with their mixtures in all different media (aqueous/50 mmol∙kg^–1^ NaCl/500 and 1000 mmol∙kg^–1^ NH_2_CONH_2_) were given in Table 6. In aqueous solution, the value of *N*_agg_ individual drug AMH was found in well approval with formerly reported value in addition to the *N*_agg_ value of individual gemini 16-E2-16 was also found to be in fine consent with the exposed value [1,30,34]. An assessment from Table 6 demonstrates that the *N*_agg_ values for mixed systems of constituent (AMH and 16-E2-16) in the absence/existence of salt/urea have been obtained to be more than both individual constituents showing the encouraging constituents interaction inside mixed micelles in every mixed system escorting to probably micelles formation of bigger size. It is also clear from the table that with an increase in *α*_1_ of gemini in mixed systems, *N*_agg_ value increases in every studied medium. In the presence of an electrolyte, the *N*_agg_ value of singular components increases together with their mixed systems whereas in the existence of 500 mmol.kg^–1^ NH_2_CONH_2_ the value of *N*_agg_ reduces for singular as well as mixed systems from aqueous systems. In a similar fashion, with enhance in the amount of NH_2_CONH_2_, the value of *N*_agg_ further decreases in the entire systems. An electrolyte is identified to diminish the electrostatic repulsion amongst the charged head groups causing larger values of aggregation number. In contrast, NH_2_CONH_2_ is recognized to boost the repulsions among head groups of constituents, as a result *N*_agg_ of studied systems reduces. The decrease in value of *N*_agg_ in the presence of urea was previously observed by other researchers also [75]. In actual fact, seeing that molecules of urea are around two and a half times larger than water particles, for this reason urea replaces several H_2_O particles from the associated structure solvation layer.

**Table 6:** Aggregation number (*N*_agg_) and other related parameters for AMH-16-E2-16 mixtures in different media at temperature *T* = 298.15 K and pressure *p* = 0.1 MPa^a^.

*α*_1_	*N*_agg_	*I*_1_/*I*_3_	*K*_sv_ x 10^−4^	*D*_exp_	*D*_ideal_
Aqueous system					
0	22	1.57	1.54	45.23	
0.1	28	1.40	13.94	31.71	28.94
0.3	34	1.34	9.66	27.06	28.12
0.5	39	1.38	5.08	29.83	28.61
0.7	48	1.32	4.78	25.21	28.91
0.9	55	1.30	3.26	23.69	28.95
1	21	1.33	4.65	25.98	
50 mmol∙kg^-1^ NaCl					
0	36	1.42	1.08	33.82	
0.1	39	1.38	21.09	29.94	23.91
0.3	46	1.31	12.66	24.50	23.79
0.5	54	1.30	9.32	23.61	24.20
0.7	62	1.30	6.29	23.41	24.26
0.9	71	1.28	5.81	23.91	23.95
1	28	1.29	4.94	22.89	
500 mmol∙kg^-1^ urea					
0	19	1.59	1.65	46.24	
0.1	23	1.41	11.94	32.33	26.73
0.3	29	1.33	10.06	25.68	26.67
0.5	35	1.31	7.42	24.22	26.86
0.7	41	1.31	6.80	24.75	27.48
0.9	49	1.32	5.18	24.97	26.79
1	18	1.31	4.84	24.75	
1000 mmol∙kg^-1^ urea					
0	16	1.63	1.74	48.02	
0.1	19	1.42	26.31	33.08	25.80
0.3	24	1.35	8.99	27.59	25.54
0.5	30	1.30	7.03	23.51	26.39
0.7	35	1.33	4.51	26.11	26.88
0.9	42	1.35	4.02	26.95	25.98
1	14	1.31	12.41	24.31	

^a^Standard uncertainties (*u*) are *u*(*T*) = 0.20 K, *u*(*NaCl*) = 1 mmol∙kg^-1^, *u*(*urea*) = 2 mmol∙kg^-1^ and *u*(*p*) = 5 kPa (level of confidence = 0.68). Relative standard uncertainties (*u*_*r*_) are *u*_*r*_(*N*_agg_) = ±4%, *u*_*r*_(*I*_1_/*I*_3_) = ±3%, *u*_*r*_(*K*_sv_) = ±3% and *u*(*D*_exp_/*D*_ideal_) = ±4%.

#### Micropolarity (*I*_*1*_*/I*_*3*_)

To further confirm the fluorescence outcomes, we have also carried out micropolarity investigations by means of a probe such as pyrene (PR). In the case of pure pyrene, the intensity of the fluorescence peak is less, which proposes that pyrene has confined itself in the closeness of the hydrophobic association. The molecules of PR show five very separate emission bands amongst 350 and 400 nm (Fig 7); moreover, the ratio of emission intensity of the first (*I*_1_ at 373 nm) to the third (*I*_3_ at 384 nm) bands, *I*_1_/*I*_3_, is characteristically regarded as a polarity evaluation of the microenvironment in the region of the pyrene area [76]. The micropolarity (*I*_1_/*I*_3_) of pure constituents along with their mixture in different ratios has been estimated by integration of the micellar system of two constituents well beyond their respective *cmc* values in all studied media (water, NaCl as well as NH_2_CONH_2_). The PR solubilization in the micelles causes the microenvironment of the studied solution to change and fluorescence emission spectra provide valuable information regarding the micellization happening in all different studied media (water, salt as well as urea). If the *I*_1_/*I*_3_ value was found to be more than 1, that shows that in the system pyrene occurs in a polar atmosphere, however, if the value of *I*_1_/*I*_3_ was found to less than 1, that demonstrates the pyrene is in nonpolar atmosphere [77].

The values of *I*_1_ and *I*_3_ are decreased through increase in quencher (CC) concentration. Characteristic values of *I*_1_/*I*_3_ for H_2_O, C_7_H_8_, CH_3_OH, C_2_H_5_OH as well as C_6_H_12_ are 1.84, 1.04, 1.33, 1.23 as well as 0.6 respectively.^78^ The entire studied pure as well as their mixtures micropolarity (*I*_1_/*I*_3_) values in all different media are shown in Table 6. In our case, all values of micropolarity (*I*_1_/*I*_3_) were obtained to be more than 1, indicating that PR exists in a more polar district as compared with the AMH, 16-E2-16 plus their mixtures [78]. Consequently, it is probably supposed that PR is solubilized in the constituency of the palisade layer of the associated structure. For the mixed systems of constituents in all different studied media, it is found that through increasing *α*_1_ of 16-E2-16 in the system of AMH–16-E2-16 mixture the value of micropolarity (*I*_1_/*I*_3_) decreases, showing that the increase of hydrophobic interactions between constituent of the mixed micelles accordingly atmosphere experienced through PR is less polar in nature in conformity by means of the reduced values of *cmc* in increase in *α*_1_ of gemini. In the case of urea for individual AMH solution, the value of *I*_1_/*I*_3_ was attained to be more as compared with an aqueous solution, which signifies that the polarity of the PR environment rises due to presence of NH_2_CONH_2_ while in the presence of salt their value decreases (Table 6). Urea increases the surface area per head groups, causing the incorporation of more H_2_O particles into the palisade layer. Thus, the polarity experienced by means of the probe increases. On the contrary, the increased surface area per head group encourages the PY to put back superficial of the micelle that cause more polar environment.

The above outcomes can be additionally clarified on account of quenching of pyrene solution by CC. The Stern–Volmer binding constants (*K*_sv_) for the individual along with mixed amphiphiles systems were found by means of Eq (25) to estimate the strength of the hydrophobic environment wherein the probe as well as quencher is situated:

$$\frac{I_{0}}{I_{1}}=1+K_{SV}[Q]$$ (25)

The estimated *K*_sv_ are listed in Table 6. The Stern–Volmer quenching constant (*K*_sv_) was computed from the plot of *I*_0_/*I* against [Q]. The greater the solubility of the PR along with CC, the higher possibility of *K*_sv_ value (Table 6). The values of *K*_sv_ for the case of a mixture of constituents in different ratios are beyond the individual AMH micelles showing the additional hydrophobic atmosphere in the mixed systems as compared with pure AMH in all different media (aqueous/electrolyte/urea) again in conformity with lower *cmc* value of mixed system as compared with *cmc* value. Table 6 also reveals that the *K*_sv_ values are not much worthy to a large level as *K*_sv_ values were obtained small.

The investigated dielectric constant (*D*_exp_) of the solution mixed systems was estimated through the subsequent Eq (26) [79]:

$$\frac{I_{1}}{I_{3}}=1.00461+0.01253D_{exp}$$ (26)

Experimental dielectric constant (*D*_exp_) for individual drug AMH micelles was obtained to be high as compared with gemini surfactant 16-E2-16 in all studied solvents (Table 6). Table 6 also clarified that *D*_exp_ values do not show any definite trend and *D*_exp_ values for singular constituents plus their mixtures were achieved between 23 to 48 in all studied solvents that were nearer the *D*_exp_ value of CH_3_OH and CH_5_OH, again proving that the atmosphere of PY is polar.

The ideal dielectric constant (*D*_ideal_) of a mixture of solution was computed by employing Eq (27) [79]:

$$D_{ideal}=\sum D_{i}X_{i}$$ (27)

It is apparent from the data that the *D*_exp_ values were reasonably unlike from the values of *D*_ideal_ showing that mixed micelles produced via AMH and 16-E2-16 mixtures in all various solvents show several attractive interactions (Table 6).

### FT-IR measurements

**Fig 8:**
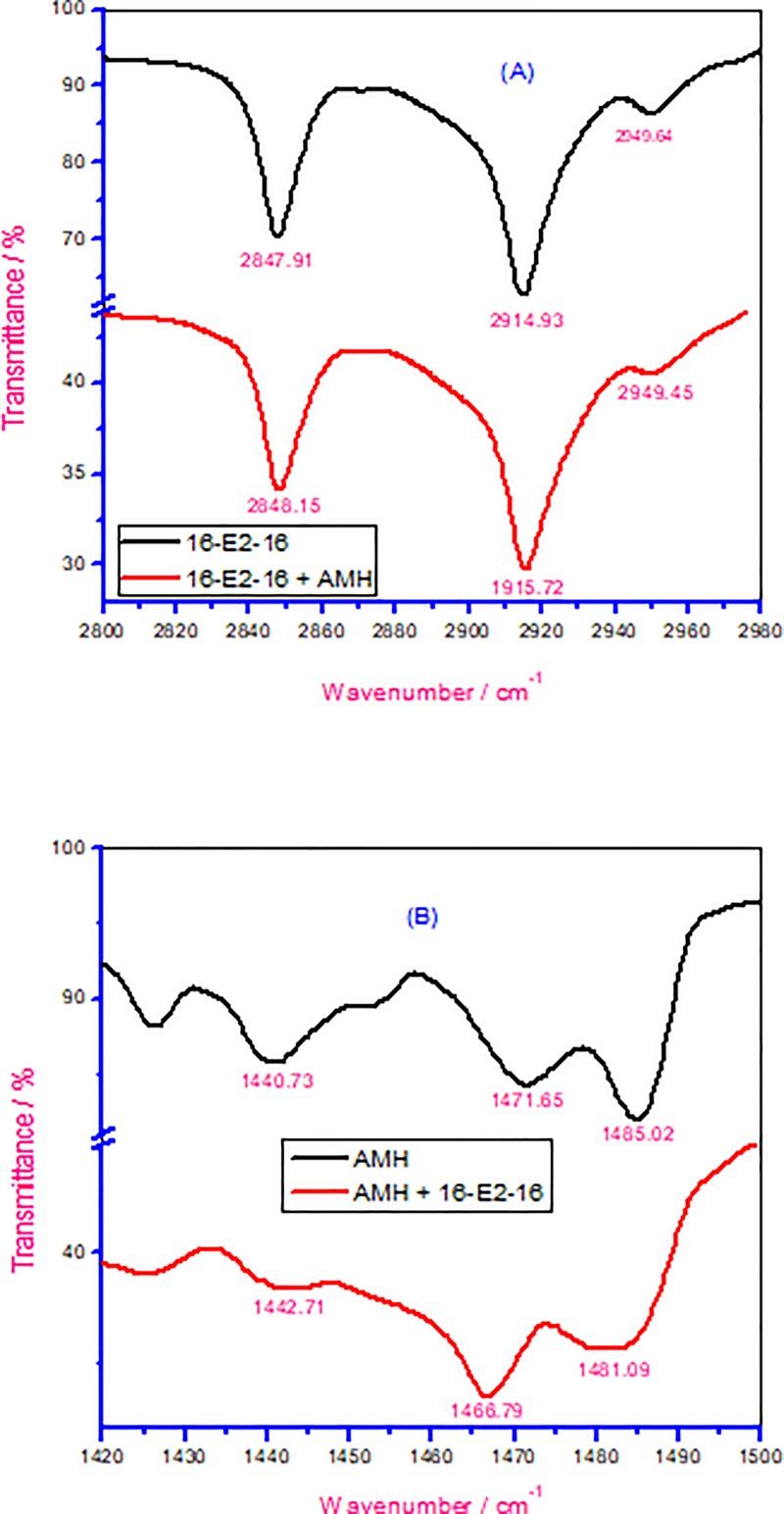
FTIR spectra of (A) 16-E2-16 in absence and presence of AMH in equal ratio and (B) AMH in absence and presence of 16-E2-16 in equal ratio.

This method is employed for examining the interaction among constituents of mixed micelles and vesicles [80,81]. Headgroup as well as the hydrophobic portion of molecules frequencies give knowledge regarding the structural transformation in the assembly of molecules [82]. Fig 8(A) shows the spectra of gemini in the absence in addition to the presence of AMH in the region between 2980 and 2800 cm^–1^. 16-E2-16 displays bands as a result of the symmetric (*v*_s_ C–H) stretching of methylene chain at 2847.91 cm^–1^ and asymmetric (*v*_as_ C–H) stretching at 2914.93 and 2949.64 cm^–1^. In the presence of AMH (drug) in the solution of 16-E2-16, the symmetric stretching of C–H bands of 16-E2-16 moved to a higher frequency (2848.15 cm^–1^) viewing the interaction between the studied constituents. The asymmetric stretchings of C–H bands of 16-E2-16 were also shifted to new frequencies (from 2914.93 to 2915.72 cm^–1^ and 2949.64 to 2949.45 cm^–1^). The change in frequency owing to interaction among constituents was found to be small and reproducible [83].

Fig 8(B) depicts the spectra of AMH along with AMH–16-E2-16 mixtures from 1500 cm^–1^ to 1400 cm^–1^. The currently employed drug contains the cationic nitrogen to which two methyl groups and one methylene are directly attached. The possible interaction of drug and gemini will shift the C–H bending or stretching frequency in the head group of the drug. Pure drug spectra reveal the bending of C–H bands of methyl (-CH_3_) and methylene (-CH_2_-) groups at 1485.62, 1471.65, and 1440.73 cm^–1^. In the occurrence of 16-E2-16 in the solution of AMH the bending of C–H bands were shifted from their primary position (1485.62, 1471.65, and 1440.73 cm^–1^) to 1481.09, 1466.79 and 1442.71 cm^–1^, respectively. The shifting in the frequency through the addition of 16-E2-16 points out the interparticle interaction among constituents. Overall shifts in stretching as well as bending frequency of all studied solution mixtures suggest the interaction among the studied components.

## Conclusions

Detailed tensiometric and fluorescence studies of the interaction between the antidepressant drug AMH and the green gemini surfactant, 16-E2-16 were executed in three dissimilar solvents (water/electrolyte/urea (two different concentrations)) at 298.15 K. Owing to the existence of salt *cmc* values of the studied system in various ratios were found to decrease whereas in the existence of NH_2_CONH_2_ their value rises. In the presence and absence of electrolyte/urea the interfacial as well as micellar conduct in the mixed systems were examined where *cmc* values of mixtures were found below ideal *cmc* (*cmc*^id^), that demonstrates the attractive interaction amongst the studied constituents (drug and gemini). The $X_{1}^{Rub}$ values in all cases were achieved negative, also viewing interaction in solution mixture and their negative value increases through raising the mole fraction of 16-E2-16 owing to an increase in hydrophobic interactions. The *N*_agg_ value for individual in addition to all studied mixtures in various solvents increases through raising the proportion of 16-E2-16 in solution mixtures. The experimentally evaluated and calculated apparent dielectric constants in all studied solutions were found to be less than their ideal value owing to attractive interactions within the micelle. Shifts in stretching along with bending frequencies of studied systems were found, suggesting an interaction among the constituents.
